# Early versus late intramedullary nailing for traumatic femur fracture management: meta-analysis

**DOI:** 10.1186/s13018-018-0856-4

**Published:** 2018-06-28

**Authors:** Ayman El-Menyar, Mohammed Muneer, David Samson, Hassan Al-Thani, Ahmad Alobaidi, Paul Mussleman, Rifat Latifi

**Affiliations:** 1 Department of Surgery Clinical Research Unit, Westchester Medical Center Health Network, Valhalla, New York USA; 2 Trauma Surgery, Clinical Research, Hamad General Hospital, Doha, Qatar; 3 Clinical Medicine, Weill Cornell Medical School, Doha, Qatar; 4 Department of Surgery, Hamad General Hospital, Doha, Qatar; 5 Department of Surgery, Trauma and Vascular Surgery, Hamad General Hospital, Doha, Qatar; 6 Department of Surgery, Orthopedic Surgery, Al Wakrah Hospital, Doha, Qatar; 7 Distributed eLibrary, Weill Cornell Medical School, Doha, Qatar; 8 Department of Surgery, Westchester Medical Center Health Network and New York Medical College, Valhalla, New York USA

**Keywords:** Meta-analysis, Intramedullary nailing, Femur fracture, Quality assessment, Orthopedic, Trauma

## Abstract

**Introduction:**

There is no consensus yet on the impact of timing of femur fracture (FF) internal fixation on the patient outcomes. This meta-analysis was conducted to evaluate the contemporary data in patients with traumatic FF undergoing intramedullary nail fixation (IMN).

**Methods:**

English language literature was searched with publication limits set from 1994 to 2016 using PubMed, Scopus, MEDLINE (OVID), EMBASE (OVID), Web of Science, and Cochrane Central Register of Controlled Trials (CENTRAL). Studies included randomized controlled trials (RCTs), prospective observational or retrospective cohort studies, and case-control studies comparing early versus late femoral shaft fractures IMN fixation. Variable times were used across studies to distinguish between early and late IMN, but 24 h was the most frequently used cutoff. The quality assessment of the reviewed studies was performed with two instruments. Observational studies were assessed with the Newcastle-Ottawa Quality Assessment Scale. RCTs were assessed with the Cochrane Risk of Bias Tool.

**Results:**

We have searched 1151 references. Screening of titles and abstracts eliminated 1098 references. We retrieved 53 articles for full-text screening, 15 of which met study eligibility criteria.

**Conclusions:**

This meta-analysis addresses the utility of IMN in patients with FF based on the current evidence; however, the modality and timing to intervene remain controversial. While we find large pooled effects in favor of early IMN, for reasons discussed, we have little confidence in the effect estimate. Moreover, the available data do not fill all the gaps in this regard; therefore, a tailored algorithm for management of FF would be of value especially in polytrauma patients.

## Background

Traumatic femur fracture (FF) continues to be associated with a considerable rate of morbidity and mortality [1]. However, these unfavorable outcomes could be contributed to several factors such as patient age and stability, mechanism of injury, comorbidities, associated injuries, and the time and approach of the management. Diaphyseal FF is mostly result of high-energy trauma and its incidence ranges from 9.9 to 12 for every 100,000 persons/year [2, 3]. Prior data showed that the average patient age is 25 years and around 60% of injuries occur in men. The considerable energy required to cause many of these fractures often provokes injuries in other structures, above all is the ipsilateral hip and knee and they often go undiagnosed [2–4].

The treatment of choice for diaphyseal FF is internal fixation with intramedullary nail (IMN) stabilization; it achieves correct alignment and high rate of bone healing with low complications rate and early limb mobility [5–7]. However, the optimal timing for internal fixation remains controversial [8–13].

The concept of early stabilization of long bone fractures including FF was established many years ago. It was suggested to provide early total care for patients of polytrauma associated with head injury. This approach was aiming to have lesser pulmonary complications and reduced length of hospital stay [14–17]. However, it was recently accepted to perform a minimal intervention surgery (i.e., damage control approach) that temporarily fixes FF until the patient general condition allows definitive management. There is no consensus regarding the effect of early versus late fixation of FF on the clinical outcomes. We conducted this meta-analysis with extensive statistical assessment to evaluate the contemporary data in patients with traumatic FF undergoing early versus late IMN, in different clinical settings. We sought to explore the literatures to know whether the concurrent meta-analysis able to fill the gaps in the nailing management of FF.

## Methodology

### Search strategy and registration

English language literature was searched with publication limits set from 01 June 1994 to 31 June 2016 in the following scholarly databases: PubMed, Scopus, MEDLINE (OVID), EMBASE (OVID), Web of Science, and Cochrane Central Register of Controlled Trials (CENTRAL). Additionally, searches were conducted in the gray literature in order to retrieve relevant unpublished scholarly materials.

Focused terms (subject headings, synonyms, etc.) utilized in the searches were reflective of the broad terms of “intramedullary nailing,” “femur fracture,” and “shaft.” This study was registered it in the PROSPERO registry (CRD 42017057866). This report follows the Preferred Reporting Items for Systematic Reviews and Meta-Analyses (PRISMA) statement [18].

### Study types

Studies included randomized controlled trials (RCTs), prospective observational or retrospective cohort studies, and case-control studies comparing early versus late femoral shaft fractures IMN fixation. Variable times were used across studies to distinguish between early and late IMN, but 24 h was the most frequently used cutoff.

### Participant types

We included studies with adult patients, any sex, and with no restriction on inclusion of ethnicities or patients with co-morbidities.

### Data extraction and management

Data were extracted by a single reviewer, confirmed by two other reviewers, and entered into EndNote. Information included authorship, publication year, methodology of the study, population, intervention, timing of the surgery, and relevant outcome measures.

### Inclusion criteria

*Included traumatic* patients with femur shaft fractures that were treated by reamed antegrade IMN, all patients below 70 years and above 14 years and single and bilateral femur fractures are both included.

### Exclusion criteria

*Included patients* below 14 years or above 70 years, patients with trochanteric, neck, or distal FF, treated FF by temporal external fixation followed by definitive IMN, patients died within 24 h of admission, and pathological FFs.

### Outcomes

Both clinical outcomes and surrogates were included, i.e., mortality, length of hospital stay, ICU length stay, any complications, bed sore, wound infection, sepsis, organ failure, all pulmonary complications, acute respiratory distress syndrome (ARDS), pneumonia, deep vein thrombosis (DVT), pulmonary embolism, and fat embolism .

### Study quality assessment

The quality assessment of the reviewed studies was performed with two instruments. Observational studies were assessed with the Newcastle-Ottawa Quality Assessment Scale [19]. Randomized controlled trials were assessed with the Cochrane Risk of Bias Tool [20].

### Statistical analysis

Unadjusted odds ratios (ORs) were extracted from individual studies. When articles also reported ORs adjusted for confounders, the magnitude of confounding was computed as the difference between the unadjusted OR and the adjusted OR divided by the unadjusted OR, expressed as a percentage. Meta-analyses were carried out using the inverse variance weighted random effects method. Heterogeneity was assessed with the *Q* statistic and *I*^2^. Forest plots contain individual study ORs and 95% confidence intervals (CIs), along with pooled ORs which were considered statistically significant if the 95% confidence interval (CI) excluded the null value of 1.0, equivalent to a *p* value < 0.05. Funnel plots were produced as a graphic technique to assess potential publication bias. Egger’s regression tests of publication bias were also conducted and were considered significant if the *p* value for slope was < 0.05. Meta-analytic forest plots and funnel plots were produced with Review Manager 5.3 (Cochrane Collaboration, Copenhagen, Denmark). Tests for publication bias were performed with STATA 9.2 (StataCorp, College Station, TX).

## Results

### Study selection

**Fig. 1:**
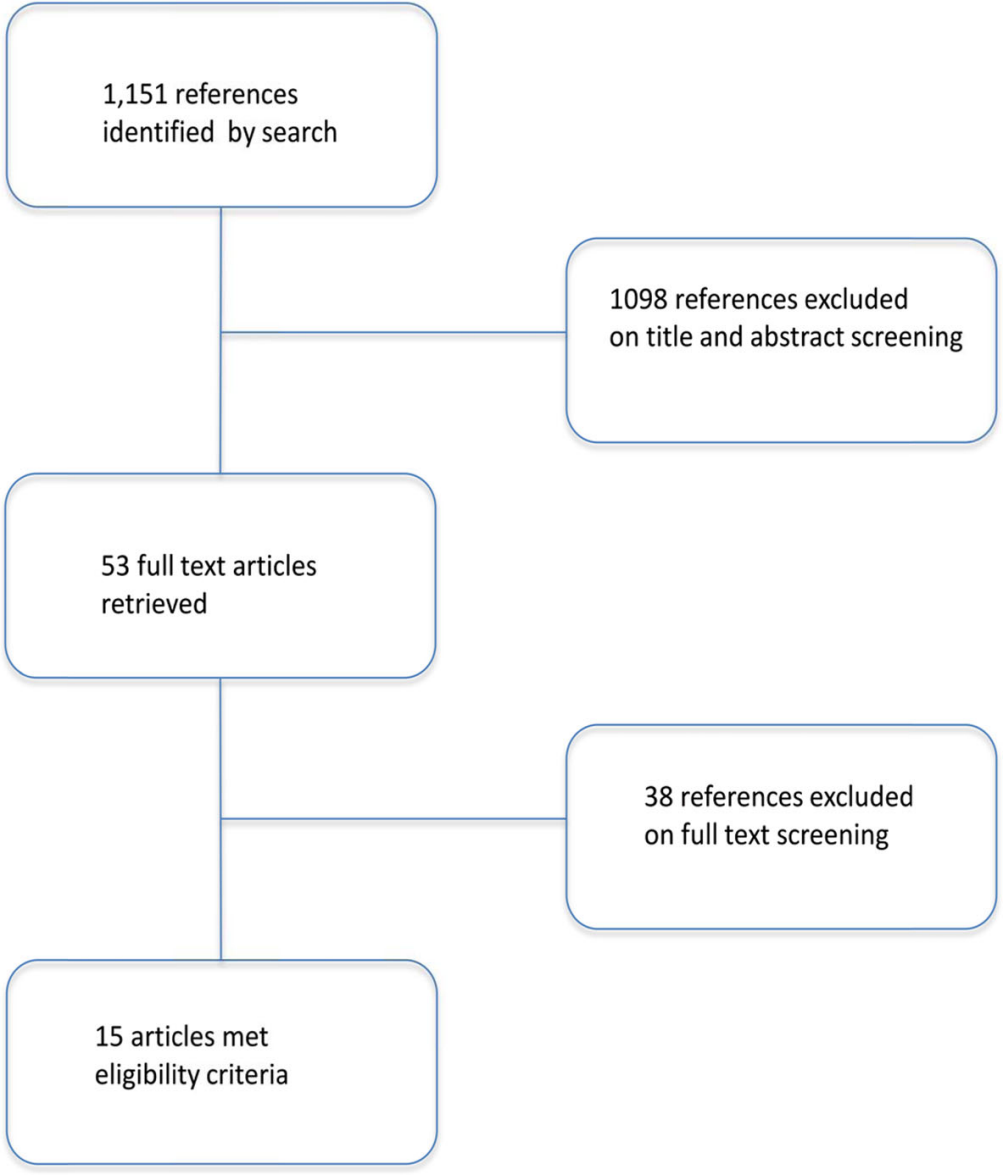
Search screening flow diagram

Search of PubMed, EMBASE, and Cochrane Central Register of Controlled trials identified 1151 references. Screening of titles and abstracts eliminated 1098 references. We retrieved 53 articles for full-text screening, 15 of which met study eligibility criteria (Fig. 1).

#### Study quality assessment

Two studies were RCTs, and 13 were retrospective cohort studies. All cohort studies earned favorable (star) ratings on these items: (1) representativeness of the exposed (early IMN) cohort; (2) selection of the non-exposed (late IMN) cohort; (3) ascertainment of exposure; (4) assessment of outcome; (5) sufficient length of follow-up and adequacy of follow-up of cohorts. No cohort study clearly stated whether participants were free of the outcomes of interest at the start of the study. Only two cohort studies [21, 22] addressed comparability of cohorts on the basis of the design or analysis, earning two stars. Harvin et al. [21] conducted multivariable logistic regression modeling in which IMN performed < 24 h or was not adjusted for injury severity score (ISS) and revised trauma score (RTS). Morshed et al. [22] constructed a marginal structural model using inverse probability of treatment-weighting and adjustment for 10 potential confounding variables. Another cohort study [23] described use of multivariate regression analysis to control for age, ISS, and type and severity of associated injuries; however, the article did not report adjusted OR estimates and is thus rated unfavorably on comparability of cohorts.

Of the two RCTs, the trial by Pape et al. [24] was generally rated more favorably. This study was rated as low risk of bias on these items: selection bias–random sequence generation; selection bias–allocation concealment; attrition bias–incomplete outcome data; and reporting bias–selective reporting. Despite favorable ratings for selection bias, this study observed several statistically significant differences on baseline characteristics and used multivariable regression models that controlled for ISS and RTS. This trial was rated as high risk of bias for detection bias—blinding of outcome assessment and was rated as unclear bias on performance bias—blinding of participants and personnel. Pape adjusted for ISS, RTS, and head injury severity in their analyses. At baseline, early IMN and late IMN groups differed significantly on these variables, in addition to the new injury severity score (NISS), and abbreviated injury scale (AIS) head. The RCT by Bone et al. [25] was rated as high risk for performance bias and detection bias and low risk for attrition bias and reporting bias. It was rated as unclear bias for both selection bias items.

#### All complications

Two retrospective cohort studies reported results on any and all complications [23, 30]. Neither study addressed comparability of groups in design or analysis. A high degree of statistical heterogeneity was observed (*I*^2^ = 98%). The summary estimate of odds of any complication between early and late IMN was not statistically significant (pooled OR = 1.28, 95% CI 0.08, 20.84).

#### Pulmonary complications

**Fig. 2:**
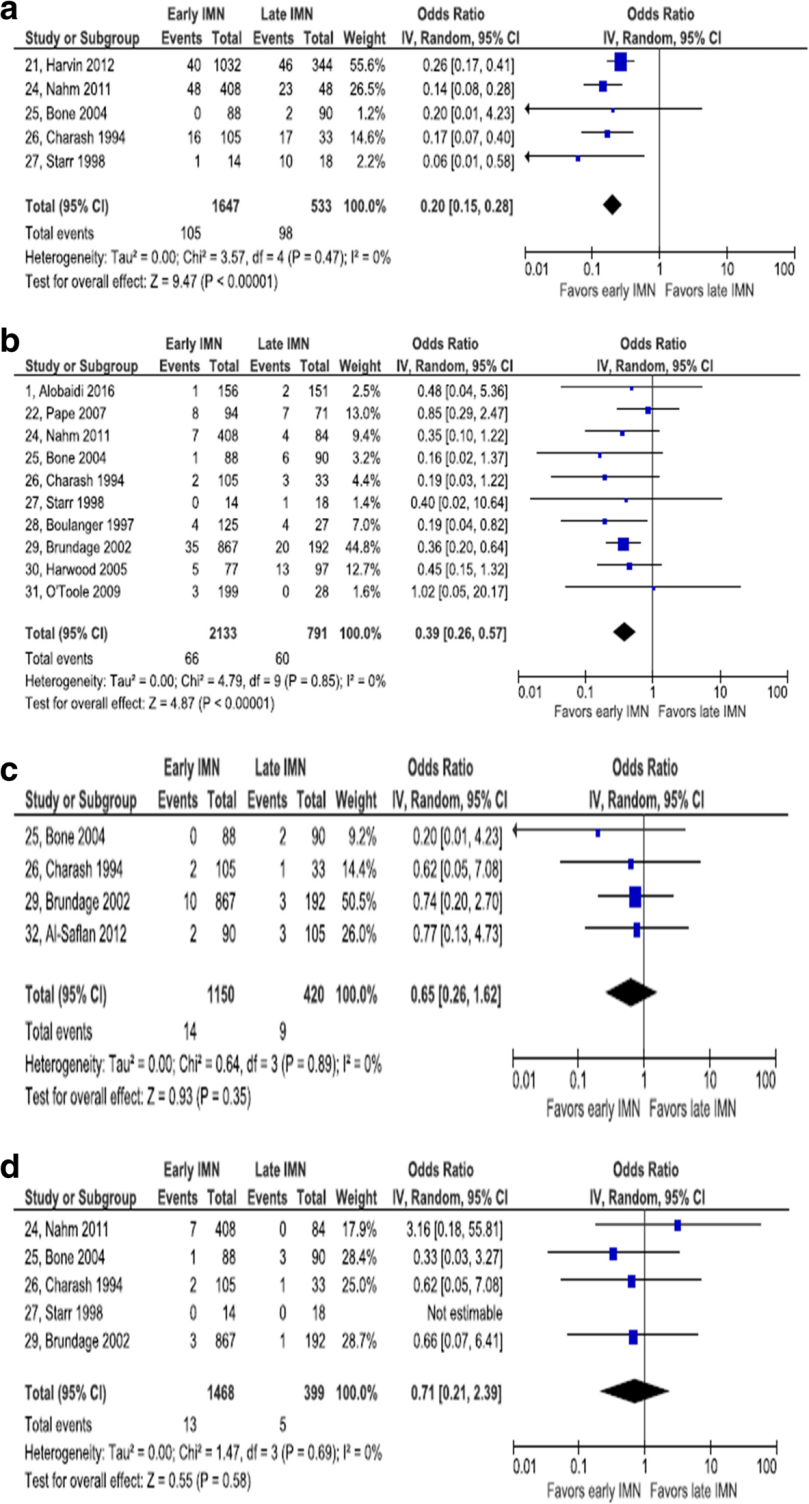
Forest plot of pulmonary complications. **a** Pulmonary complications. **b** Acute respiratory distress syndrome. **c** Fat embolism. **d** Pneumonia

One RCT and four retrospective cohort studies contributed to this meta-analysis [21, 24, 25–27]. The RCT was rated as high risk of selection bias. One of the cohort studies addressed comparability in the analysis [21], but only the unadjusted data are included in this meta-analysis. Of the four studies that reported the composite outcome, pulmonary complications, all included both pneumonia and pulmonary embolism in the outcome definition. Nahm et al. also included ARDS. Charash et al. [26] added fat embolism, while Bone et al. [25] added abnormal blood gas levels. These five studies collectively enrolled 2180 patients. No statistical heterogeneity was observed (*I*^2^ = 0%). Early IMN was associated with a lower odds of pulmonary complications, compared with late IMN (pooled OR = 0.20, 95% CI 0.15, 0.28). The study by Harvin et al. [21] reported both an unadjusted OR and an adjusted OR. The magnitude of confounding was calculated as − 64.6%, moving the point estimate from 0.26 to 0.43; statistical adjustment yielded a weaker association between early IMN and pulmonary complications (Fig. 2).

#### Acute respiratory distress syndrome

Two RCTs and 8 retrospective cohort studies reported on ARDS [1, 22, 24–31]. Only the RCT by Pape et al. [22] reported adjusted estimates of the OR, but the meta-analysis includes only the unadjusted OR from this study. No other studies attempted to control for confounding either by design or analysis. The aggregate number of patients in these 10 studies is 2924. No statistical heterogeneity was observed (*I*^2^ = 0%). Early IMN was associated with a lower odds of ARDS, compared with late IMN (pooled OR = 0.39, 95% CI 0.26, 0.57). Pape et al. [22] presented both unadjusted and adjusted ORs. The magnitude of confounding was − 20.0%, enough to null the impact of early IMN: the unadjusted OR, used in pooling, was 0.85, whereas the adjusted OR was 1.02.

#### Pneumonia

One RCT and six retrospective cohort studies presented data on the incidence of pneumonia [22, 24–27, 29, 30]. The Pape et al. [22] RCT provided the OR adjusted for ISS and RTS; however, only the unadjusted OR is included in this meta-analysis. Together, these 7 studies included 2238 patients. There was a low degree of statistical heterogeneity among these studies (*I*^2^ = 31%). Early IMN was associated with a lower odds of pneumonia, compared with late IMN (pooled OR = 0.37, 95% CI 0.26, 0.54). By comparing unadjusted and adjusted ORs, the RCT in this set of studies showed a very large magnitude of confounding: − 186.9%. By controlling for two confounding variables the point estimate OR value changed from 0.48 to 1.39. Adjustment changed not only the magnitude of the association, but also reversed the direction.

#### Fat embolism

One RCT and three retrospective cohort studies gave data for the occurrence of fat embolism [25, 26, 29, 32]. None of the observational studies adjusted for potential confounders. The total of participants across studies was 1570. No statistical heterogeneity was observed (*I*^2^ = 0%). There was no statistically significant association between timing of IMN and odds of fat embolism. The pooled OR was 0.64 and the 95% CI was 0.26 to 1.62.

#### Pulmonary embolism

One RCT and four retrospective studies reported data for pulmonary embolism [24–27, 29], but there were zero events in both early and late groups in one study, making estimation of the OR impossible, so that study [27] was excluded. No observational study adjusted the OR for confounders. Summed across studies, the total number of patients was1867. No statistical heterogeneity was observed (*I*^2^ = 0%). There was no statistically significant association between timing of IMN and odds of pulmonary embolism. The pooled OR was 0.71 and the 95% CI was 0.21 to 2.39.

#### Deep vein thrombosis

**Fig. 3:**
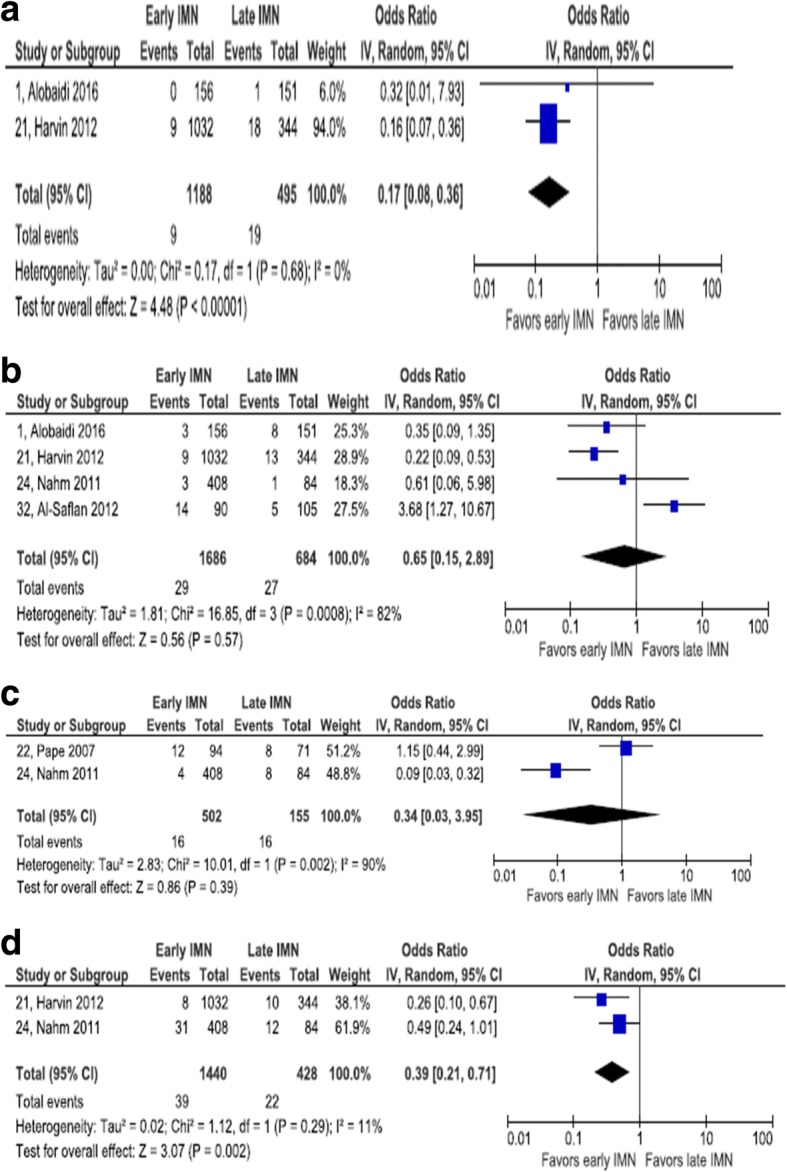
Forest plot of cutaneous, infectious, and vascular complications. **a** Decubitus ulcer. **b** Wound infection. **c** Deep vein thrombosis. **d** Pulmonary embolism

Two retrospective cohort studies provided evidence on DVT risk [21, 24]. The Harvin et al. study [21] was controlled for ISS and RTS. A total of 678 patients participated in these studies. Statistical heterogeneity among these studies is low (*I*^2^ = 11%). Compared with late IMN, early IMN is associated with a statistically significant reduction in the odds of DVT (pooled OR = 0.39, 95% CI 0.21, 0.71) (Fig. 3).

#### Multiorgan failure

One RCT and two retrospective cohort studies collected data on multiorgan failure [22, 24, 30]. The RCT adjusted for ISS and RTS. The aggregate number of patients in these two studies was 831. There was a minor degree of statistical heterogeneity (*I*^2^ = 30%). There was a statistically significant association between timing of IMN and multiorgan failure: pooled OR = 0.38, 95% CI 0.16, 0.90. The magnitude of confounding in the RCT was − 11.6%, enough to change a weak nonsignificant effect favoring early IMN to a weak nonsignificant effect favoring late IMN.

#### Wound infection

Four retrospective cohort studies reported on wound infection. None addressed confounding in the analysis [1, 21, 24, 32]. A total of 2370 patients were included in these four studies. Considerable statistical heterogeneity was observed in this set of studies (*I*^2^ = 82%). There was no statistically significant association between timing of IMN and wound infection: pooled OR = 0.65, 95% CI 0.15, 2.89.

#### Sepsis

One RCT and one retrospective cohort study reported on the risk of sepsis after IMN [22, 24]. The retrospective cohort study did not report outcomes adjusted for potential confounding, while the RCT did report adjusted outcome measures. The magnitude of confounding in the RCT was − 13.9%, indicating a slightly stronger estimate of an effect favoring late IMN after adjustment. A high degree of statistical heterogeneity among these two studies was observed (*I*^2^ = 90%). The pooled OR (0.34) did not reach statistical significance (95% CI 0.03, 3.95).

#### Decubitus ulcer

Two retrospective cohort studies addressed the incidence of decubitus ulcers [1, 21]. Neither addressed confounding in the analysis. Between these two studies, 1683 patients were enrolled. No statistical heterogeneity was observed (*I*^2^ = 0%). There was a statistically significant association between early IMN and reduced odds of decubitus ulcer occurrence: OR = 0.17, 95% CI 0.08, 0.36.

#### Mortality

**Fig. 4:**
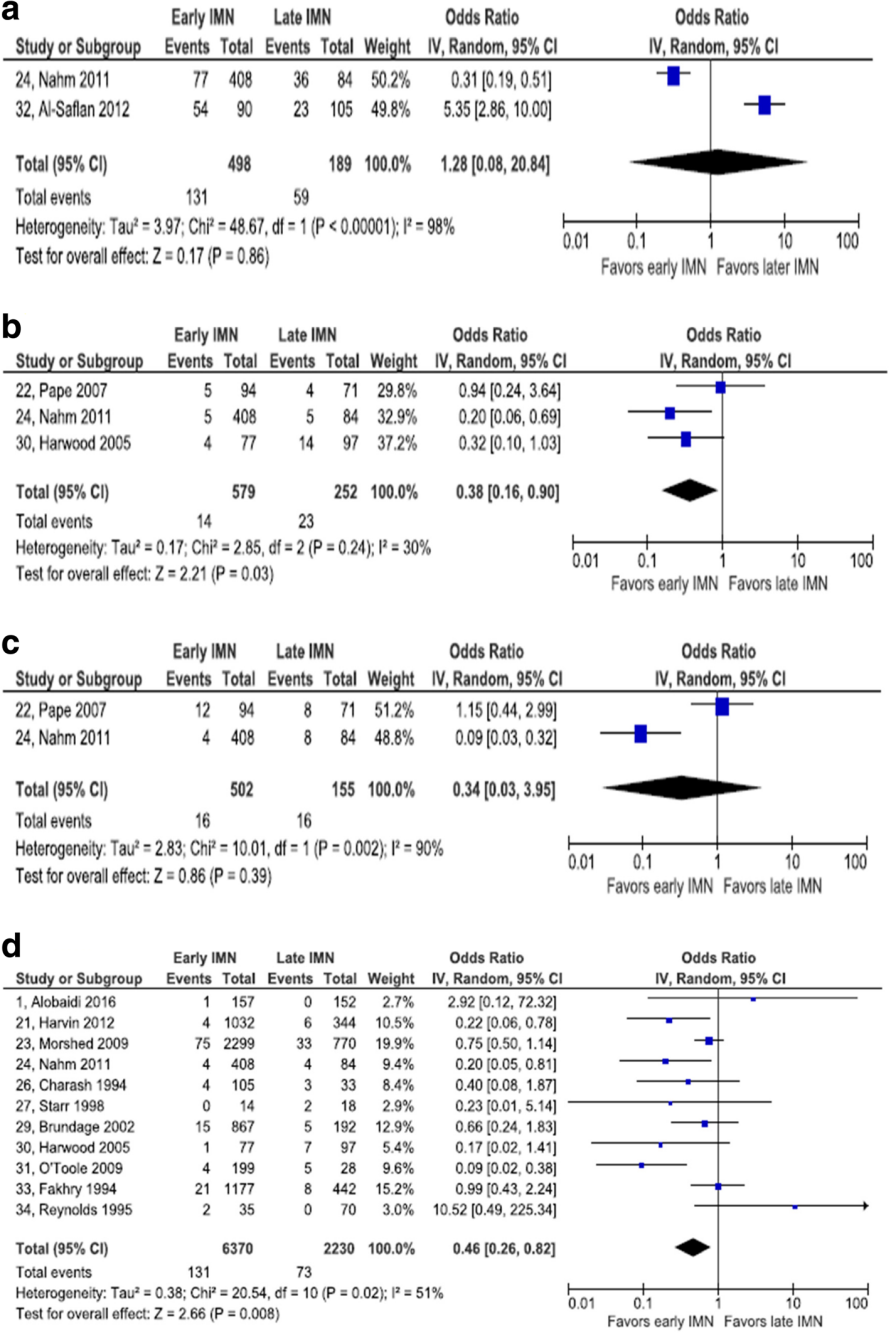
Forest plot of other complications. **a** Any complications. **b** Sepsis. **c** Multiorgan failure. **d** Mortality

Eleven retrospective cohort studies included data on mortality [1, 21, 23, 24, 26, 27, 29–31, 33, 34]. None reported ORs adjusted for confounders. The total number of patients was 8600. There was a moderate degree of statistical heterogeneity among these studies (*I*^2^ = 51%). The association between early IMN and reduced mortality achieved statistical significance: OR = 0.46, 95% CI 0.26, 0.82 (Fig. 4).

## Discussion

**Fig. 5:**
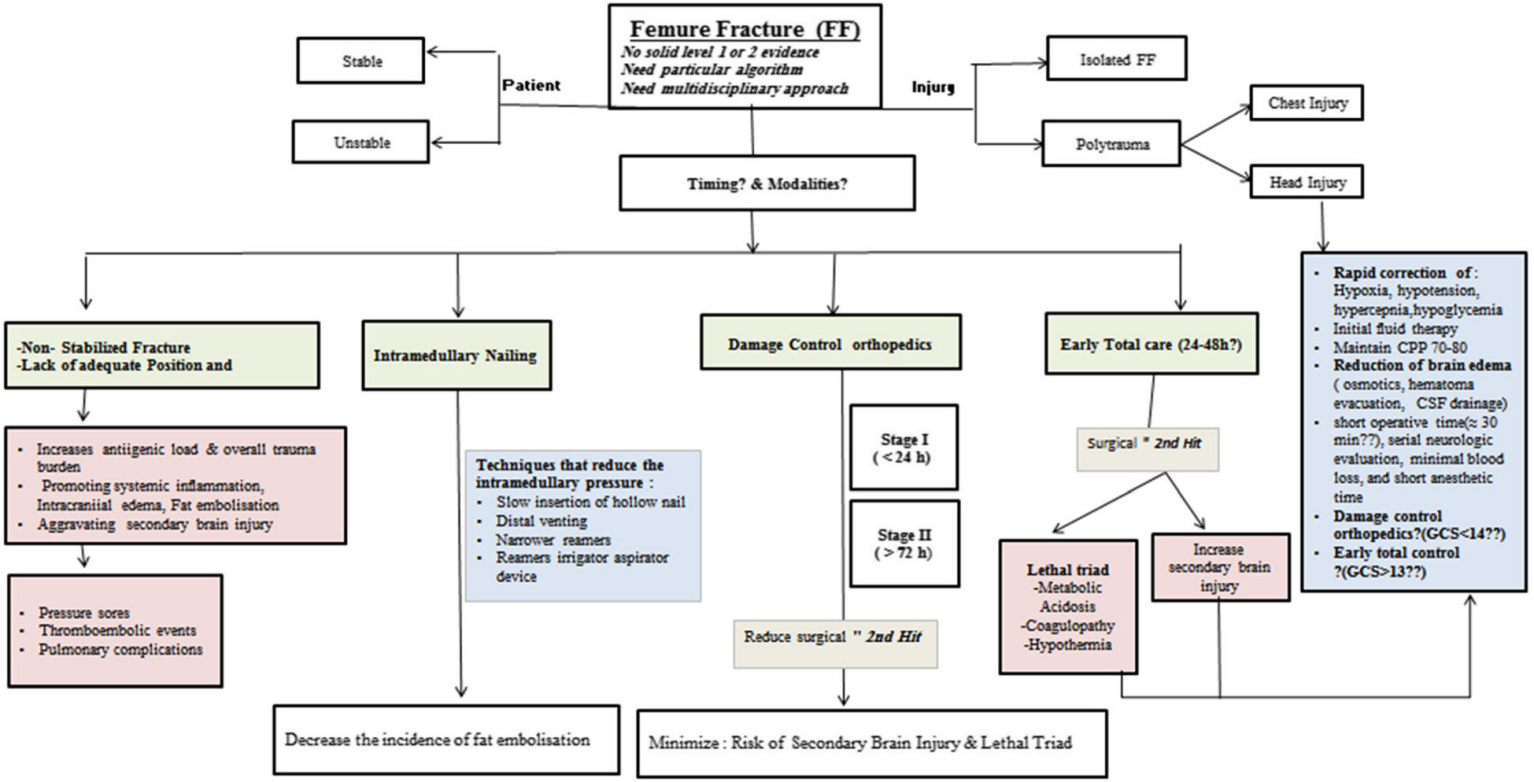
A proposed algorithm for the management of femur fracture based on the contemporary evidence in the literature

In general, the timing of surgical management in trauma patients should attain maximum benefit of surgery and minimum or nil complications. Time to intervene in orthopedic surgery has ultimate prognostic impact; however, the optimum timing is still debatable in traumatic FF [35]. In long bone and pelvic fracture, it has been shown that early surgical fixation is associated with better cardiac function and pulmonary performance, respectively [36, 37]. Of note, FF is often a high-energy injury and also might have associated thoracic [17, 25] or head injuries [15, 38–40]. Moreover, internal fixation with IMN has physiologic consequences; therefore, a lot of considerations and a particular algorithm should be in the decision-makers mind [15]. Figure 5 shows a proposed algorithm for the management of FF (shaft) in adult patients based on the contemporary evidence in the literature.

In 2016, Liu et al. published a meta-analysis that addressed the impact of early IMN in patients with severe chest trauma; however, the authors have done their search up to the year 2011 only [41]. The present meta-analysis sheds more light on this debate (early versus late IMN in FF patients) in different clinical settings up to the mid of 2016.

Among the outcomes of IMN that were pooled in the present analysis, we found statistically significant results for any pulmonary complication, ARDS, pneumonia, decubitus ulcers, multiorgan failure, DVT, and mortality, while results were not statistically significant for pulmonary embolism, fat embolism, wound infection, sepsis, and any complication.

For pulmonary injury, it was reported that early IMN fixation of femoral shaft fractures could contribute to an additional respiratory damage and may trigger ARDS in the presence of chest injury, particularly in borderline patients [24]. However, we found that early IMN has lower odds of pulmonary complications, ARDS, and pneumonia than late IMN fixation. Therefore, in some studies, it was suggested that pulmonary complications could be related to the chest injury itself rather than the methods of FF fixation in polytrauma patients [16, 24]. However, reamed nailing performed within the initial 24 h was found to have a potentially negative effect on the lung and should be avoided in the presence of chest trauma. Pape et al. [42, 43] reported a sustained inflammatory response only after intramedullary femoral instrumentation that was done within the first 24 h. In such high-risk trauma patients, it is thought that the damage control orthopedic surgery approach is a wise option to avoid such complications. Weninger et al. [44] evaluated 45 patients with severe thoracic trauma and FF stabilized with unreamed IMN within the first 24 h and found that the rates of ARDS, multiorgan failure, and mortality were not negatively influenced by early unreamed IMN. The patients’ general condition also has its own word for the method and outcome after IMN in polytrauma cases.

This meta-analysis did not show a significant association between the time of IMN and the occurrence of pulmonary embolism. However, Gray et al. [45] found that IMN resulted in a significantly high initial pulmonary embolic load; but there was no detectable effect on the coagulation profile, pulmonary inflammation, or mortality over the first 24 h after injury. In one study, Brundage et al. [29] reported 17 (1.2%) cases with fat embolism (13 in the operative and 4 in non-operative group) with no significant association between its occurrence and the time of IMN. Also they reported five cases of pulmonary embolism (all were in the operative group and 4 of them were associated with high ISS). A prior meta-analysis of six studies founds no difference in the incidence of venous thromboembolism between early and late IMN groups [35].We could not find any information reading the use of DVT prophylaxis in the entire analyzed studies.

The relationship between early fracture and the risk of fat embolism has been addressed in few studies [46–49]. In one study, due to increased medullary canal pressure during nailing process, fat extravasation into the lung vasculature has been shown during reaming by intraoperative transesophageal echocardiogram [47]. There are some approaches like slow insertion of hollow nails, distal venting, narrower reamers, and reamer irrigator aspirator devices have been shown to reduce this intramedullary pressure [50, 51].

In our meta-analysis, patients who had early IMN were significantly associated with fewer decubitus ulcers. However, wound infection was shown to have no association with the timing of IMN. Early IMN allows patients earlier rehabilitation and mobility. Therefore, the risk to develop to decubitus ulcer is less.

There is an ongoing debate regarding the optimal temporal approach for surgical stabilization in patients with concomitant head injuries. During the first 24 h post injury, intramedullary fracture fixation, may reduce the patient’s mean arterial pressure and cerebral perfusion pressure and lead to secondary brain insults and deterioration of the neurologic state [10, 52, 53].

There is a lack of prospectively randomized trials that focus on the temporal approach of fracture fixation in multiple trauma patients with concomitant head injury [10, 13].

Jaicks et al. [39] reported on 33 patients with closed head injury (AIS score > 2) that required operative fracture fixation. They found a higher rate of intraoperative hypotension (62%) and hypoxia (11%) in the early stabilized fracture cases with a worse neurologic outcome. It has been reported that intraoperative hypotension or hypoxia explained the lower discharge GCS score in patients who underwent early fracture fixation in patients with concomitant head injuries [39, 52].

Advocates of delayed surgical management of femoral fractures in patients with concomitant head injury maintain the high risk of secondary brain insult if the aggressive treatment of fractures interferes with resuscitation or neurosurgical monitoring [10, 13]. Moreover, Townsend et al. reviewed 61 patients with moderate-to-severe blunt head injury and FF who were divided into four groups based on the timing of the orthopedic surgical correction [52]. The investigators found a significantly higher risk of intraoperative hypotension (68%) in patients who underwent definitive fracture fixation within the first 2 h of admission in comparison to 8% if fracture fixation was delayed more than 24 h. In the current meta-analysis, there were eight studies that described the management of FF in patients with head injury, of which only four studies reported the severity of head injury (head AIS > 2) [25, 31, 33, 54].

Prior reports showed that short operative time (≈ 30 min), serial neurologic evaluation, minimal blood loss, and short anesthetic time (i.e., damage control approach) are important factors to minimize secondary brain injury [15].

The present analysis shows that early IMN is associated with less mortality rate which goes in line with most of the studies; however, the association between timing of IMN and multiorgan failure was not significant.

### Limitations

In this meta-analysis, Newcastle-Ottawa Quality Assessment Scale was used to assess the methodologic quality of observational studies. Included cohort studies were rated favorably on most items. The exceptions were the selection bias item regarding demonstration that the outcome of interest was not present at the start of the study and comparability of cohorts on the basis of the design or analysis. None of the 16 cohort studies was favorable on the former and only two studies reported attempts to control for confounding in statistical analysis. The lack of control for potential confounding is a major flaw in this evidence base. In the few instances in which studies reported both unadjusted ORs and adjusted ORs, it is possible to quantify the magnitude of confounding present. Across four outcomes, adjustment nearly always resulted in moving the point estimate away from the more favorable unadjusted estimate. Thus, adjustment tended to lead to weaker estimates of association and in one case led to an estimate characterized by the opposite direction of association.

The GRADE system for assessing the strength of a body of evidence recommends starting with a level of low evidence when it is based primarily on observational studies [55]. GRADE also recommends reducing strength to very low when observational studies fail to take confounding into account in the analysis. While this meta-analysis finds large pooled effects favoring early IMN, for reasons discussed, we have little confidence in the effect estimate. So far, it seems that the available meta-analyses would not fully answer or fill the gaps in the current clinical practice.

## Conclusions

This meta-analysis addresses the utility of IMN in patients with FF based on the current evidence; however, the modality and timing to intervene remain controversial. While we find large pooled effects in favor of early IMN, for reasons discussed, we have little confidence in the effect estimate. Moreover, the available data do not fill all the gaps in this regard; therefore, a tailored algorithm for management of FF would be of value especially in polytrauma patients.

## Abbreviations

ARDS: Acute respiratory distress syndrome
DVT: Deep vein thrombosis
FF: Femur fracture
IMN: Intramedullary nailing
ISS: Injury severity scoring
RTS: Revised trauma scoring
