# Correction to: Early versus late intramedullary nailing for traumatic femur fracture management: meta-analysis

**DOI:** 10.1186/s13018-018-0884-0

**Published:** 2018-07-24

**Authors:** Ayman El-Menyar, Mohammed Muneer, David Samson, Hassan Al-Thani, Ahmad Alobaidi, Paul Mussleman, Rifat Latifi

**Affiliations:** 1 Department of Surgery Clinical Research Unit, Westchester Medical Center Health Network, Valhalla, NY USA; 2 Trauma Surgery, Clinical Research, Hamad General Hospital, Doha, Qatar; 3 Clinical Medicine, Weill Cornell Medical School, Doha, Qatar; 4 Department of Surgery, Hamad General Hospital, Doha, Qatar; 5 Department of Surgery, Trauma and Vascular Surgery, Hamad General Hospital, Doha, Qatar; 6 Department of Surgery, Orthopedic Surgery, Al Wakrah Hospital, Doha, Qatar; 7 Distributed eLibrary, Weill Cornell Medical School, Doha, Qatar; 8 Department of Surgery, Westchester Medical Center Health Network and New York Medical College, Valhalla, NY USA

## Correction

Following the publication of this article [1], the authors reported that they had submitted an incorrect version of Figs. 2, 3 and 4. They apologize for this error and the correct versions of Figs. 2, 3 and 4 with captions have been included in this Correction. There is a typographical error in the following sentence: Two retrospective cohort studies reported results on any and all complications [23, 30]. The correct version of this sentence is: Two retrospective cohort studies reported results on any complication [24, 34].

**Fig. 2:**
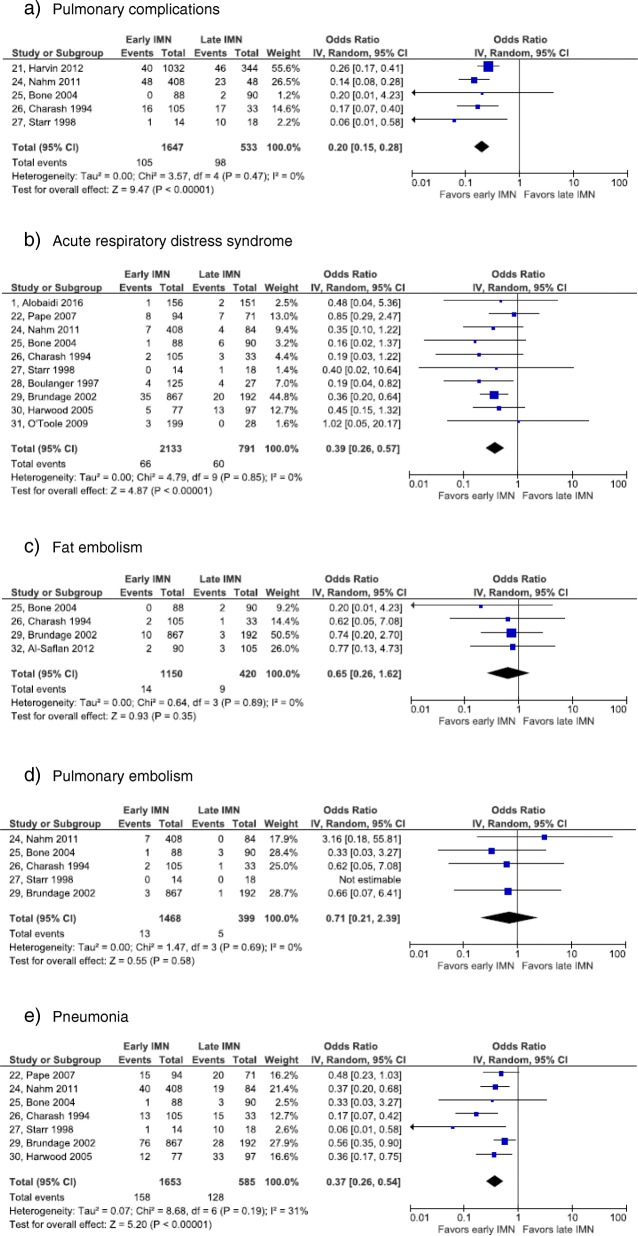
Forest plot of pulmonary complications. **a** Pulmonary complications. **b** Acute respiratory distress syndrome. **c** Fat embolism. **d** pulmonary embolism. **e** Pneumonia

**Fig. 3:**
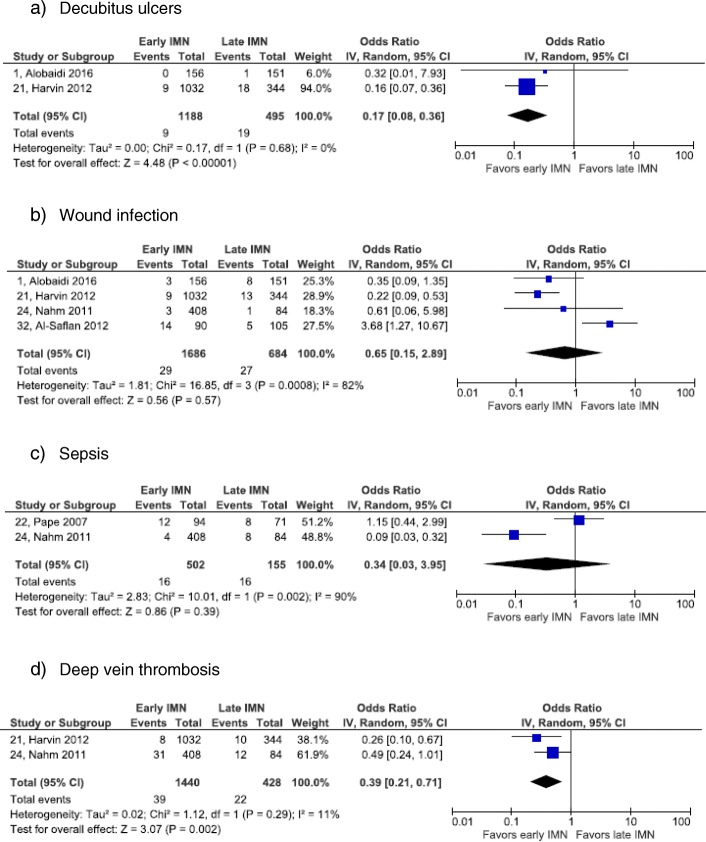
Forest plots of cutaneous, infectious and venous complications: **a** decubitus ulcers. **b** wound infection. **c** sepsis. **d** deep vein thrombosis

**Fig. 4:**
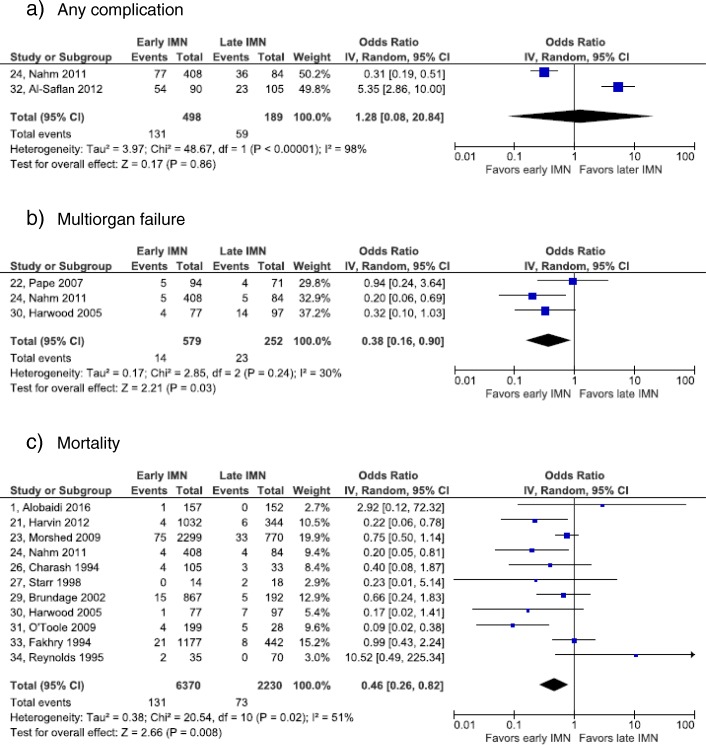
Forest plots of other complications: **a**. Any complication. **b** multiorgan failure **c.** mortality

## References

[1] El-Menyar (2018). Early versus late intramedullary nailing for traumatic femur fracture management: meta-analysis. J Orthop Surg Res.

